# Retrospective evaluation of endoscopic stenting of combined malignant common bile duct and gastric outlet-duodenum obstructions

**DOI:** 10.3892/etm.2014.1899

**Published:** 2014-08-11

**Authors:** JIANFENG YU, JIANYU HAO, DONGFANG WU, HAIBO LANG

**Affiliations:** Department of Gastroenterology, Beijing Chao-Yang Hospital, Capital Medical University, Beijing 100020, P.R. China

**Keywords:** stent, malignant gastric outlet obstruction, common bile duct obstruction, duodenal obstruction

## Abstract

Malignant dual obstruction in the common bile duct and gastric outlet-duodenum can cause difficulties in palliative treatment. The purpose of this study was to summarize our successful experience with the endoscopic stenting procedure for the palliative treatment of malignant biliary and gastric outlet-duodenum obstruction. Seventeen patients who underwent dual stenting procedures for the common bile duct and duodenum were retrospectively reviewed. The success rate of placement, palliative effect for biliary and duodenal obstruction, incidence of complication and restricture and stent patency were analyzed. Stent placement achieved a 100% success rate. Total bilirubin decreased from 263.4±62.5 to 157.6±25.1 μmol/l, direct bilirubin decreased from 233.2±66.5 to 130.9±27.7 μmol/l and alkaline phosphatase from 534.2±78.7 to 216.3±23.3 IU/l. The differences between the preoperative and postoperative results were statistically significant (P<0.01). The gastric outlet obstruction score increased significantly from 0.9±1.1 to 2.1±0.7 points (P<0.01). The general nutritional status of the patients was improved. No serious complications occurred in any of the patients, and the survival time of patients following stenting ranged between 70 and 332 days with a mean survival time of 192 days. In conclusion, our methodology for combined biliary and enteral stenting is highly effective for the palliation of malignant biliary and gastric outlet-duodenal obstruction.

## Introduction

It is not uncommon in clinical settings to encounter patients with combined malignant gastric outlet obstruction (GOO) and common bile duct obstruction (CBO). This can be caused by pancreatic cancer, cholangiocarcinoma and ampullary duodenal cancer, as well as a number of other malignant tumors (1,2). Conventional treatment is a surgical bypass procedure, which is associated with significant complications and high mortality rates (3). Therefore, since the introduction of the self-expanding metal stent (SEMS), stent placement has become the preferred option in clinical palliative management (4,5). For patients with symptoms resulting in a progressive deterioration in the quality of life and limited life expectancy, treatment is focused on palliation to ensure that quality of life is maintained (6–9). These symptoms include significant obstructive jaundice, dark urine, itching, severe nausea, vomiting, intolerance to oral feeding and abdominal pain. Endoscopic stent placement is now a standard therapy for the management of biliary strictures (10–12) and has been well accepted as clinical routine practice in malignant CBO (13–15), malignant GOO (16,17), malignant duodenal obstruction (18–20) and malignant colonic obstruction (21–24). However, few studies on dual endoscopic SEMS placement with the co-existence of GOO and CBO have been published. It is believed that, since extension of the duodenoscope to the duodenal papilla is made difficult by GOO, this practice has become unpopular in clinics. In the present study, our experience in applying endoscopic biliary and duodenal stenting in 17 patients was summarized and the techniques used to obtain a satisfactory clinical outcome were described.

## Materials and methods

### Patients

The study was approved by the Ethics Committee of Chao-Yang Hospital (Beijing, China). In total, 17 patients aged 62–87 years, including 14 males and three females, were reviewed. All exhibited GOO and CBO, and received endoscopic biliary and duodenal stent placements between January 2008 and May 2012. Fourteen of the patients had pancreatic cancer, two had cholangiocarcinoma and one had duodenal cancer, all of which were unresectable. The locations of the GOO and CBO are listed in Table I. Patients had clear symptoms of GOO and CBO prior to treatment including skin and sclera jaundice, dark brown urine, lighter stool color, vomiting and abdominal pain. GOO and CBO were confirmed by biochemical tests, computed tomography, magnetic resonance imaging and endoscopy. Improvements in oral intake were monitored using the Gastric Outlet Obstruction Scoring System (GOOSS) (6).

### Surgical procedure

Patients were fasted for three days before surgery. The stomach tube was placed for decompression when required to ensure a clear vision of the endoscope. Anisodamine (20 mg; Modern Hasen Pharmaceutical Co. Ltd., Shanghai, China) and diazepam (10 mg; Shanghai Xudong Haipu Pharmaceutical Co., Ltd., Shanghai, China) were administered intravenously for gastric motility inhibition and sedation, respectively, prior to the surgery.

**Figure 1:**
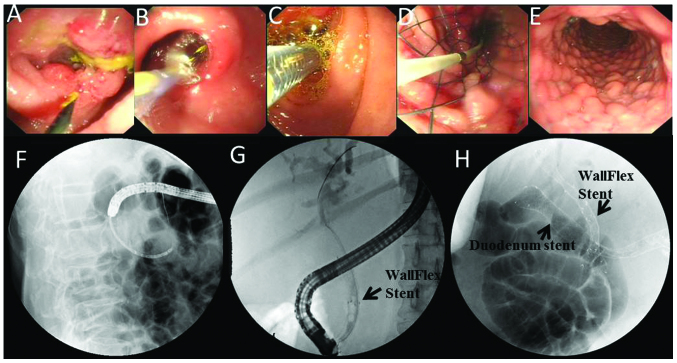
Endoscopic and cholangiographic images of malignant strictures and stent placement. (A) A guidewire was inserted through the duodenal stricture with (B) a CRE™ dilation balloon inside the duodenal stricture and (C) a stent introducer with a WallFlex™ stent to the duodenal stricture. (D) The stent was released from the introducer; (E) the duodenal stent was completely placed at the gastric outlet obstruction location; (F) a WallFlex stent was placed into the common bile duct; (G) a gastric endoscope was placed in the duodenum, through which a Jagwire^®^ guidewire was inserted into the jejunum; and (H) dual stents for the duodenal and common bile duct strictures were positioned.

Stages of the surgical procedure are shown in Fig. 1. The initial step was to establish the duodenal passage. An ultra-slim gastric endoscope (Olympus Corp., Tokyo, Japan) was placed into the gastric antrum or duodenum, reaching to the distal end of the obstruction segment. A Jagwire^®^ guidewire (Boston Scientific, Natick, MA, USA) was then inserted through the endoscope chamber into the jejunum (Fig. 1A). The obstruction size was measured while the endoscope was pulled out. If the ultra-slim endoscope failed to pass through the stricture segment, the guidewire was inserted from the biopsy channel. A double-lumen catheter was then introduced along the guidewire to the distal end of the stricture. Diatrizoate (30%; Hansen Pharmaceutical Co., Ltd., Hunan, China) was injected through the catheter while the catheter was pulled to the proximal end of the stricture for the obstruction length measurement. The guidewire was then retained and the ultra-slim gastric endoscope or catheter was withdrawn, followed by insertion of an electronic duodenoscope (TJF240; Olympus Corp.) along the guidewire to reach the duodenal bulb, and then insertion of a CRE™ dilation balloon (Boston Scientific) to gradually dilate the duodenal stricture under direct visualization (Fig. 1B). The ideal expansion would be ≥13.5 mm in diameter to allow the duodenoscope to pass through.

Following a successful dilation of the duodenum, the duodenoscope was then moved forward into the descending section of the duodenum. Papillary intubation and cholangiography were performed, and a guidewire was re-introduced through the catheter to the distal end of the bile duct stricture. With the guidewire, an introducer carrying a biliary WallFlex™ stent (Boston Scientific) with an appropriate length was placed into the common bile duct (Fig. 1C and F).

The ultra-slim gastric endoscope was then re-introduced into the duodenum, through which a guidewire was inserted into the jejunum. The ultra-slim endoscope was replaced with an ordinary gastric endoscope carrying a duodenal stent (Boston Scientific) into the stricture (Fig. 1D and G). One end of the stent was placed on the opening of the pyloric side (Fig. 1E and H).

### Postoperative evaluation and treatment

Antibiotics were administered for three days after the surgery as a preventive measure. Serum amylase levels were checked 2 h after the surgery and, if normal, a liquid diet was allowed. Postoperative observation was focused on signs and symptoms for upper gastrointestinal complications including hyperamylasemia and upper gastrointestinal bleeding and perforation. No special diet formula was administered if the obstruction symptoms disappeared. Total bilirubin, direct bilirubin, alkaline phosphatase, GOOSS score and nutritional indicators, including triglycerol, albumin and hemoglobulin, were measured on day 7. Telephone follow-up was performed for migration, occlusion, possible procedure- or device-related complications and survival time.

### Statistical analysis

The statistical analysis was performed using IBM SPSS statistical software version 21.0 (IBM-SPSS, Cary, NC, USA). Paired differences prior and subsequent to surgery were assessed with a Student’s t-test. P<0.05 was considered to indicate a statistically significant difference.

## Results

### Postoperative observations

Seventeen stenting procedures were performed successfully on patients with both GOO and CBO in the Beijing Chao-Yang Hospital (Beijing, China) between January 2008 and May 2012 with a success rate of 100%. Adequate clinical palliation was observed, and details are listed in Table II. Improvements in the incidence of spontaneous clinical manifestation were evidenced by the immediate disappearance of vomiting and abdominal pain, as well as the gradual disappearance of jaundice. Serum amylase levels were checked at 2 h after the surgery. The majority of the patients exhibited normal levels, with the exception of three patients who developed transient hyperamylasemia, and who recovered without intervention within 72 h. Minor bleeding occurred only in one case in the duodenal stricture during balloon dilation, and ceased following application of an ice-cold adrenaline saline spray. Biochemical measurements were taken after seven days. Average total bilirubin decreased by 40%, direct bilirubin decreased by 44% and alkaline phosphatase decreased by 60% postoperatively (Table II). The GOOSS score increased from 0.9±1.1 points preoperatively to 2.1±0.7 points postoperatively. These differences were statistically significant (P<0.01). Nutritional status was found to be significantly improved following the surgery (Table II).

### Follow-up

No stent migration was found during the follow-up period. Occlusion occurred in one case at the distal end of the stent after three months due to tumor infiltration or compression. A three-cavity jejunal feeding tube was placed for decompression and nutrition. Restricture was found at the biliary location in two cases due to tumor invasion, which caused obstructive jaundice; this symptom was relieved following percutaneous transhepatic drainage. The mean survival time of patients was 192 days ranging between 70 and 332 days.

## Discussion

Surgical treatment, including biliary-enteric anastomosis or gastrojejunostomy, has been traditionally applied in cases of GOO and CBO. However, such traumatic surgery has been associated with serious complications and a failure to increase patient survival rate. For patients unable to tolerate surgery, decompression and enteral feeding tubes have typically been provided as alternative methods (22,25–27). In recent years, this traditional approach has been gradually replaced by other minimally invasive approaches. SEMS placement is an approach that has demonstrated advantages and cost effectiveness over the traditional treatments (4,5). In the cases of CBO and GOO, however, few reports have been published regarding dual stents for palliation of these two obstructions. Certain studies used oral duodenal stenting in combination with percutaneous transhepatic biliary metal stent implantation (28,29). The endoscopic procedure with dual stenting should be the first choice for its minimal invasiveness, lower tissue damage, and fewer postoperative complications. Dual stents were successfully installed in the present study in 17 patients with GOO and CBO without any significant complications. The data demonstrated that the procedure was safe and effective, and the quality of life of the patients was significantly improved following the procedure.

Several key elements should be addressed for a successful result in this dual stenting procedure. Firstly, it is very important to establish an endoscopic duodenal corridor. Conventionally, it has been considered that endoscopes cannot reach the duodenal papilla due to GOO. It is difficult for the endoscope to pass through the obstruction without causing complications, such as bleeding, perforation or tumor rupture. The CRE wire-guided balloon dilation catheter has gained traction to overcome this problem by applying controlled radial expansion (30–33). Guided by the wire, the axis of the balloon can be kept in parallel with the axis of the luminal cavity, thus preventing the tip of the balloon catheter from piercing into the duodenal wall or tumor, which could cause bleeding or perforation. Therefore, the balloon can be negotiated across the stricture, during which appropriate pressure can be gradually applied to avoid any abrupt shearing force, and the center of the balloon can be always kept at the center of the stricture.

Usually, endoscopic retrograde cholangiopancreatography (ERCP) following CRE balloon dilation for the duodenum would be difficult since i) the duodenum space is smaller than the normal duodenum, even subsequent to dilation; ii) the normal structure of the duodenal papilla may have been damaged by tumor invasion; and iii) balloon dilatation may cause tissue edema that further increases the difficulty of recognizing the papilla. Due to the smaller space inside the duodenum, the experience in the present study was that the exposed metal stents inside the duodenum should be ≤10 mm to avoid damage of the contralateral duodenal wall. In addition, since patients often experienced prolonged biliary obstruction that caused the accumulation of higher pressure in the bile duct, extra precaution was taken when the angiography was performed, including the withdrawal of a small portion of bile prior to injection of the contrast reagent, and ensuring only necessary volumes of the contrast reagent were injected.

Finally, duodenal stenting requires precise positioning. A drawback force would help position the stent while it was released from the introducer to keep it within the stricture segment. In addition, great care should be taken to avoid the guidewire penetrating through the mesh of the exposed portion of the biliary stent. X-ray-guided endoscopic guidewire placement cannot precisely determine the guidewire position. Application of the ultra-slim endoscope is much more convenient for guiding as well as observing the procedure of the stent placement.

Although endoscopic biliary metal stenting and duodenal stenting exhibit a minimally invasive character, complications can still be an occasionally serious problem. Major complications include hyperamylasemia, post-ERCP pancreatitis, cholangitis, biliary fistula, bleeding, perforation and migration. Three patients in the present study were observed to develop transient hyperamylasemia (17.6%) and one patient exhibited duodenal bleeding following dilation (5.9%), which is similar to what has been reported in the literature (34).

The major long-term complication is stent restricture due to tumor infiltration. Duodenal stent restricture can be relieved by a new stent placement in the original duodenal stent. For common bile duct stent restricture, it is not appropriate to perform ERCP-related processing since the duodenal stent blocks the duodenal papilla. One of the options would be to perform percutaneous transhepatobiliary puncture for biliary drainage, while inserting the guidewire through the drainage into the distal end of duodenum. An expandable balloon of 8–19 mm with the guidewire could expand the duodenal stent mesh so that the mesh could be opened wide enough to allow biliary stent placement. The balloon could also be used to assist the complete expansion of the biliary stent. No clear interference was observed between the duodenal and biliary stents, which is consistent with previously reported results (29,35).

One of the parameters monitored postoperatively in the present study was the nutritional status of the patients. The data showed that levels of triglycerol, albumin and hemoglobulin improved following the surgery, indicating that the nutritional status of the patients did not continue deteriorating. Furthermore, the improvement in the GOOSS score demonstrated the benefit of the procedure for the quality of life of the patients.

In conclusion, endoscopic dual stent placement for combined GOO and CBO is a safe and effective procedure that can greatly improve the quality of life and nutritional status of patients. However, the recanalization of GOO and CBO does not address the cause of the malignant obstruction. With regard to the continued growth and spread of tumors, it would be interesting to explore the possibilities of preventing restricture by local chemotherapy or biotherapy with stenting (36).

## Figures and Tables

**Table I:** Demographic summary and location of obstructions.

Parameter	Value
Gender (n)	
Male	14
Female	3
Age (years)	76.6±6.5
Malignancy (n)	
Pancreatic cancer	14
Cholangiocarcinoma	2
Primary duodenal carcinoma	1
Location of CBO (n)	
Distal common bile duct	10
Mid CBO	2
Proximal and mid CBO	4
Papilla	1
Location of GOO (n)	
Pylorus	2
Above the major papilla	5
At the major papilla	8
Under the major papilla	1

Total n=17. CBO, common bile duct obstruction; GOO, gastric outlet obstruction. Age is expressed at the mean ± standard deviation.

**Table II:** Comparison of patients prior and subsequent to the stenting surgery.

Item	Prior to surgery	Following surgery	P-value
Clinical manifestations (n)			
Vomiting	17	1	
Abdominal pain	17	2	
Jaundice	17	4	
Dark urine	17	0	
Serological tests			
Total bilirubin (μM/l)	263.4±62.5	157.6±25.1	<0.01
Direct bilirubin (μM/l)	233.2±66.5	130.9±27.7	<0.01
Alkaline phosphatase (IU/l)	534.2±78.7	216.3±23.3	<0.01
Triglycerol (mmol/l)	0.61±0.07	0.68±0.08	<0.01
Albumin (g/l)	32.08±2.15	33.13±1.54	<0.01
Hemoglobulin (g/l)	135.60±14.35	145.30±8.44	<0.01
GOOSS score	0.9±1.1	2.1±0.7	<0.01

GOOSS, Gastric Outlet Obstruction Scoring System. Results are expressed as the mean ± standard deviation.

## References

[1] Khullar SK, DiSario JA (1996). Gastric outlet obstruction. Gastrointest Endosc Clin N Am.

[2] Johnson CD, Ellis H (1990). Gastric outlet obstruction now predicts malignancy. Br J Surg.

[3] Oida T, Mimatsu K, Kano H, Kawasaki A, Kuboi Y, Fukino N, Kida K, Aramaki O, Amano S (2011). Palliative enteric bypass for malignant gastric outflow obstruction after pancreaticoduodenectomy in early recurrent pancreatic cancer. Hepatogastroenterology.

[4] Kozarek RA, Ball TJ, Patterson DJ (1992). Metallic self-expanding stent application in the upper gastrointestinal tract: caveats and concerns. Gastrointest Endosc.

[5] Topazian M, Ring E, Grendell J (1992). Palliation of obstructing gastric cancer with steel mesh, self-expanding endoprostheses. Gastrointest Endosc.

[6] Adler DG, Baron TH (2002). Endoscopic palliation of malignant gastric outlet obstruction using self-expanding metal stents: experience in 36 patients. Am J Gastroenterol.

[7] Piesman M, Kozarek RA, Brandabur JJ, Pleskow DK, Chuttani R, Eysselein VE, Silverman WB, Vargo JJ, Waxman I, Catalano MF, Baron TH, Parsons WG, Slivka A, Carr-Locke DL (2009). Improved oral intake after palliative duodenal stenting for malignant obstruction: a prospective multicenter clinical trial. Am J Gastroenterol.

[8] Tempero M, Arnoletti JP, Ben-Josef E, Bhargava P, Casper ES, Kim P, Malafa MP, Nakakura EK, Shibata S, Talamonti M, Wang H, Willett C (2007). Pancreatic adenocarcinoma. Clinical Practice Guidelines in Oncology. J Natl Compr Canc Netw.

[9] van Hooft JE, Uitdehaag MJ, Bruno MJ, Timmer R, Siersema PD, Dijkgraaf MG, Fockens P (2009). Efficacy and safety of the new WallFlex enteral stent in palliative treatment of malignant gastric outlet obstruction (DUOFLEX study): a prospective multicenter study. Gastrointest Endosc.

[10] Andersen JR, Sørensen SM, Kruse A, Rokkjaer M, Matzen P (1989). Randomised trial of endoscopic endoprosthesis versus operative bypass in malignant obstructive jaundice. Gut.

[11] Artifon EL, Sakai P, Cunha JE, Dupont A, Filho FM, Hondo FY, Ishioka S, Raju GS (2006). Surgery or endoscopy for palliation of biliary obstruction due to metastatic pancreatic cancer. Am J Gastroenterol.

[12] Speer AG, Cotton PB, Russell RC, Mason RR, Hatfield AR, Leung JW, MacRae KD, Houghton J, Lennon CA (1987). Randomised trial of endoscopic versus percutaneous stent insertion in malignant obstructive jaundice. Lancet.

[13] Chen YZ, Wang JH, Feng BS (2011). Endoscopic biliary stenting for malignant biliary obstruction in 124 cases. Zhonghua Xiao Hua Nei Jing Za Zhi.

[14] Luigiano C, Ferrara F, Cennamo V, Fabbri C, Bassi M, Ghersi S, Consolo P, Morace C, Polifemo AM, Billi P, Ceroni L, Alibrandi A, D’Imperio N (2012). A comparison of uncovered metal stents for the palliation of patients with malignant biliary obstruction: nitinol vs. stainless steel. Dig Liver Dis.

[15] Varadarajulu S, Tutuian R, Gostout C, Kozarek R, Wilcox CM, Cotton PB (2004). Efficacy of the Za self-expandable metal stent for palliation of malignant biliary obstruction. J Clin Gastroenterol.

[16] Larssen L, Medhus AW, Hauge T (2009). Treatment of malignant gastric outlet obstruction with stents: an evaluation of the reported variables for clinical outcome. BMC Gastroenterol.

[17] Yao LQ, Zhong ZS (2007). Application progress of endoscopic metal stents for treatment of gastric outlet obstruction. Zhonghua Xiao Hua Nei Jing Za Zhi.

[18] Chen YX, Li GH, Zhou XJ, Lv NH (2010). Endoscopic stenting combined with X-ray monitoring for the treatment of malignant duodenal obstruction: analysis of 9 cases. Zhonghua Xiao Hua Nei Jing Za Zhi.

[19] Johnston SD, McKelvey ST, Moorehead RJ, Spence RA, Tham TC (2002). Duodenal stents for malignant duodenal strictures. Ulster Med J.

[20] Li DM, Li RX, Hou W (2008). Endoscopic metal stenting of malignant gastroduodenal obstruction: efficacy observation. Zhonghua Xiao Hua Nei Jing Za Zhi.

[21] Cho YK, Kim SW, Lee BI, Lee KM, Lim CH, Kim JS, Chang JH, Park JM, Lee IS, Choi MG, Choi KY, Chung IS (2011). Clinical outcome of self-expandable metal stent placement in the management of malignant proximal colon obstruction. Gut Liver.

[22] Scott-Mackie P, Morgan R, Farrugia M, Glynos M, Adam A (1997). The role of metallic stents in malignant duodenal obstruction. Br J Radiol.

[23] Tominaga K, Maetani I, Sato K, Shigoka H, Omuta S, Ito S, Saigusa Y (2012). Favorable long-term clinical outcome of uncovered D-weave stent placement as definitive palliative treatment for malignant colorectal obstruction. Dis Colon Rectum.

[24] Zhong YS, Yao LQ, Xu JM (2010). Self-expanding metallic stents for acute obstruction of the proximal colorectal cancer. Zhonghua Xiao Hua Nei Jing Za Zhi.

[25] Sankarankutty AK, Mente ED, Cardoso NM, Castro-E-Silva O (2013). T-tube or no T-tube for bile duct anastomosis in orthotopic liver transplantation. Hepatobiliary Surg Nutr.

[26] Farooq A, Patel R, Sorefan N, Ammori BJ (2004). Laparoscopic exclusion gastroenterostomy for palliation of gastric outlet obstruction secondary to recurrent cholangiocarcinoma. Hepatogastroenterology.

[27] Feretis C, Benakis P, Dimopoulos C, Georgopoulos K, Milas F, Manouras A, Apostolidis N (1996). Palliation of malignant gastric outlet obstruction with self-expanding metal stents. Endoscopy.

[28] Akinci D, Akhan O, Ozkan F, Ciftci T, Ozkan OS, Karcaaltincaba M, Ozmen MN (2007). Palliation of malignant biliary and duodenal obstruction with combined metallic stenting. Cardiovasc Intervent Radiol.

[29] Zhang XQ, Liu CW, Wang SQ (2007). Treatment of malignant biliary and duodenal obstruction using percutaneous trans-hepatic catheterization in combination with per-oral stent placement. Fang She Xue Shi Jian Bian Ji Bu.

[30] Benjamin SB, Cattau EL, Glass RL (1982). Balloon dilation of the pylorus: therapy for gastric outlet obstruction. Gastrointest Endosc.

[31] Benjamin SB, Glass RL, Cattau EL, Miller WB (1984). Preliminary experience with balloon dilation of the pylorus. Gastrointest Endosc.

[32] Kochhar R, Kochhar S (2010). Endoscopic balloon dilation for benign gastric outlet obstruction in adults. World J Gastrointest Endosc.

[33] Yusuf TE, Brugge WR (2006). Endoscopic therapy of benign pyloric stenosis and gastric outlet obstruction. Curr Opin Gastroenterol.

[34] Vlavianos P, Zabron A (2012). Clinical outcomes, quality of life, advantages and disadvantages of metal stent placement in the upper gastrointestinal tract. Curr Opin Support Palliat Care.

[35] van Groeningen CJ (1999). Intravenous and intra-arterial chemotherapeutic possibilities in biliopancreatic cancer. Ann Oncol.

[36] Fisher SB, Fisher KE, Maithel SK (2012). Molecular targeted therapy for biliary tract malignancy: defining the target. Hepatobiliary Surg Nutr.

